# Strabismus Surgery for Psychosocial Reasons—A Literature Review

**DOI:** 10.22599/bioj.352

**Published:** 2024-04-22

**Authors:** Gemma Arblaster, David Buckley, Sarah Barnes, Helen Davis

**Affiliations:** 1 Division of Ophthalmology and Orthoptics, School of Allied Health Professions, Nursing and Midwifery, University of Sheffield, UK; 2 Orthoptic Department, Sheffield Teaching Hospitals NHS Foundation Trust, UK; 3 School of Medicine and Population Health, University of Sheffield, UK

**Keywords:** strabismus, strabismus surgery, psychosocial, outcomes, quality of life, health related quality of life

## Abstract

**Introduction:**

Strabismus surgery may be undertaken for visual benefit, to improve or eliminate diplopia symptoms, or to restore or improve binocular single vision (BSV). In patients without visual symptoms or expected visual benefit, strabismus surgery may still be undertaken if the presence of strabismus causes the patient psychosocial symptoms. To evaluate strabismus surgery undertaken for psychosocial reasons, evidence of postoperative outcomes in this specific cohort is needed.

**Methods:**

A systematic search of the literature was conducted (1946–2023) to identify evidence where postoperative outcomes were reported for adult patients (age 18 years and above) who had undergone strabismus surgery for psychosocial reasons.

**Results:**

Sixty–nine papers were included in the literature review. Most sources of evidence included patients within heterogeneous cohorts of strabismus surgery outcomes, with a range of symptoms and differing surgical aims.

**Discussion:**

In adults who underwent strabismus surgery for psychosocial reasons, improved postoperative ocular alignment and/or improved health related quality of life (HRQoL) were common. Strabismus surgery outcomes appeared to be measured satisfactorily at three months postoperatively. Additional surgical outcomes, including an expanded field of vision, unexpected BSV, improved binocular summation, improved task performance and improved eye movements have been reported, but not fully investigated. There was a lack of consensus on how postoperative success should be defined and measured. A core outcome set for strabismus has been suggested and there is potential to add to the available evidence by investigating which outcome measures are most relevant to those with strabismus and psychosocial symptoms. There is a growing need for robust evidence in this specific subgroup of patients due to a lack of evidence specifically reporting postoperative outcomes in adults with strabismus and psychosocial symptoms.

## Introduction

Strabismus affects 4–5% of the population (Beauchamp et al. 2003; Goseki & Ishikawa 2017; Hashemi et al. 2017). The aim of strabismus management is to reduce or eliminate the visual and/or psychosocial symptoms caused by strabismus by realigning the eyes into a straighter position. Postoperative restoration of binocular single vision (BSV) or improved diplopia (or confusion) symptoms are considered functional aims of surgery that give the patient visual benefit. If there are no visual symptoms and no potential BSV was predicted, surgery may still be considered if the strabismus caused psychosocial symptoms (Beauchamp et al. 2003).

‘Psychosocial symptoms’ describe the impact of having strabismus on all aspects of the patient’s life. They include lower health-related quality of life (HRQoL) and quality of life (QoL) (Adams et al. 2016; Durnian et al. 2010; Fieß et al. 2020; McBain et al. 2014b; Sah et al. 2017; Wang et al. 2014) and worse self-reported visual function than other ocular diseases (Chang et al. 2015). Patients with strabismus were 10 times more likely to suffer with clinical depression or anxiety (McBain et al. 2014b). Worse depression was associated with reduced HRQoL (Hatt et al. 2014) and in children, strabismus was linked to anxiety, depression, drinking alcohol (Lin et al. 2014), and mental illness (Hassan et al. 2015; Mohney et al. 2008). Psychosocial symptoms caused by strabismus also include social phobia, social fear, social avoidance (Bez et al. 2009; Xu et al. 2012), and difficulty making eye contact and interacting with people (Ghiasi et al. 2013; Xu et al. 2012), leading to hiding strabismus from others (Ghiasi et al. 2013; Menon et al. 2002), embarrassment, negative self-esteem, and avoiding activities (Ghiasi et al. 2013). Strabismus is also reported to interfere with friendships and relationships (Burke et al. 1997), leading to feeling different, having low self-confidence, poor self-image (Satterfield et al. 1993; Xu et al. 2012), and receiving ridicule throughout life (Satterfield et al. 1993). Patients with strabismus report their psychological symptoms are not affected by diplopia or vision in the poorer eye (Ritchie et al. 2013). Patients without diplopia typically report more psychosocial symptoms (McBain et al. 2014a) and perceive their strabismus to be more noticeable and severe (Jackson et al. 2006).

Negative perceptions of strabismus have been identified in children as young as five years old (Paysse et al. 2001). Adults with strabismus were perceived negatively by others (Kothari & Joshi 2014; Olitsky et al. 1999), as significantly less intelligent, as worse at communication (Olitsky et al. 1999), as less suitable for promotion (Goff et al., 2006), and as less able in the workplace (Coats et al. 2000; Mojon-Azzi & Mojon 2009). Negative perceptions of strabismus negatively affect employment and dating opportunities (Mojon-Azzi & Mojon 2009; Mojon-Azzi et al. 2008).

Strabismus surgery for psychosocial reasons is considered low cost, relatively low risk (Bradbury & Taylor 2013; Ritchie & Ali 2019), highly cost effective (Beauchamp et al. 2005; Beauchamp et al. 2006; Fujiike et al. 2011) and beneficial for patients (Das et al. 2017; Royal College of Ophthalmologists 2016). Whilst the NHS has not withdrawn funding for strabismus surgery, some areas of England were no longer funding strabismus surgery, unless the patient has visual symptoms (such as diplopia) or proven visual benefit from treatment (such as regaining BSV). There was concern that not enough patient benefit was proven in those without expected functional visual gains from surgery (Bristol North Somerset and South Gloucestershire Clinical Commissioning Group 2019). There was therefore a need to increase the evidence of strabismus surgery outcomes specifically in patients with psychosocial symptoms (Durnian et al. 2011). This literature review aimed to evaluate the evidence of outcomes from strabismus surgery when undertaken for psychosocial reasons.

## Methods

A systematic search of the literature was undertaken. Search terms are shown in Table 1. The following databases and repositories were searched: PubMed, Scopus, Cochrane Library, NICE, PsycINFO, Web of Science, Google Scholar, the British and Irish Orthoptic Journal online and an EndNote database of non- or pre-Medline indexed sources (American Orthoptic Journal, Australian Orthoptic Journal, British Orthoptic Journal, Strabismus, Binocular Vision, Journal of AAPOS, and the Transactions of the International Orthoptic Congress, the International Strabismological Association, and the European Strabismological Association). Of specific interest were the treatment outcomes in strabismus in patients with psychosocial symptoms, but no diplopia and no demonstrable BSV. The search was purposely broadened to include larger strabismus cohorts, where surgery for psychosocial reasons may have been a subgroup.

**Table 1:** Literature search terms used.


TERMS	BOOLEAN OPERATOR	FILTERS USED

Strabismus, Adult, Surgery, Outcomes	AND	English Humans

Thyroid, Graves, Myasthenia, Nerve palsy, Myopia, Fracture, Intermittent, Duane	NOT	All adult age categories From 1/1/1946–31/12/2022

Additional search performed using the MeSH terms: Strabismus AND Surgery including the term AND psychosocial (all fields)		

Additional search performed using the terms: outcome AND functional AND eye alignment AND squint		

Initial search performed 1/11/20 (date range 1946–2020). Search updated 7/8/23 (date range 2020–2023).		


## Results

**Figure 1:**
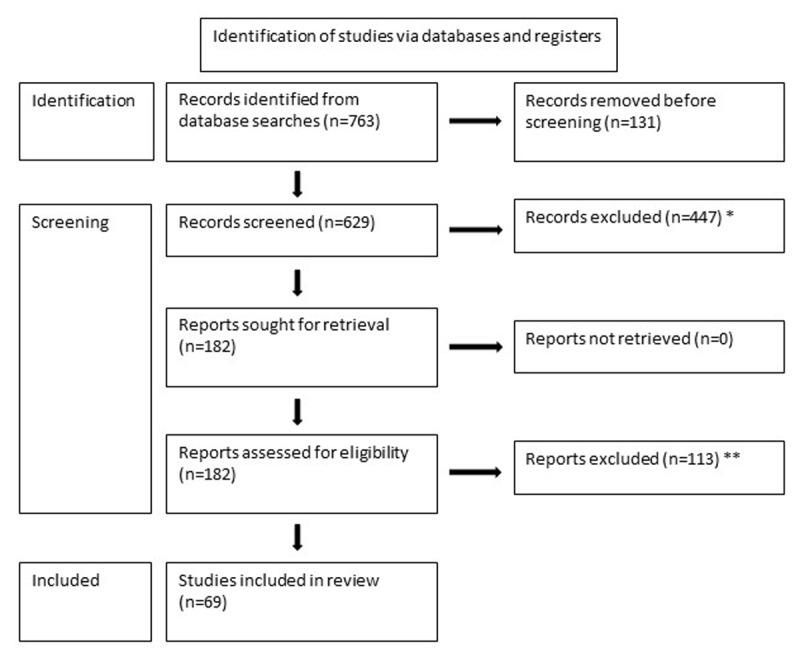
Flow chart illustrating the results of the literature search. * No automated tools were used, all records were excluded by GA. * Exclusions due to: – Strabismus surgery planned for visual benefit (to gain BSV or to eliminate diplopia), or to investigate outcomes in patients with potential BSV (for example prism adaptation to restore BSV prior to strabismus surgery).– Strabismus secondary to or associated with other aetiologies such as neurogenic palsy, mechanical condition (for example Duane syndrome), high myopia, retinal detachment, orbital fractures, congenital fibrosis of the extraocular muscles, age related distance ET (with diplopia).– Other strabismus diagnoses reported only (for example acute acquired concomitant esotropia, DVD, double elevator palsy).– Strabismus surgery outcomes in co-existing ocular pathology (for example glaucoma).– Strabismus surgery anaesthetic techniques.- Strabismus surgery but without strabismus outcome data reported or where it was unclear which patients, within a larger cohort, had undergone surgery for psychosocial reasons.– Strabismus surgery techniques and outcomes following specific vertical muscle procedures for a vertical or torsional deviation (for example Harada-lto procedure).– Intermittent strabismus or heterophoria only.– Paediatric patients only (with the following exceptions: childhood strabismus that had recurred in adulthood and childhood onset strabismus that had received the primary surgical treatment in adulthood).– Other surgical outcomes (for example refractive surgery outcomes performed in patients with strabismus).– Treatments for diplopia (with the exception of diplopia resulting from psychosocial strabismus surgery, which was included).– Slipped extraocular muscles during surgery (for example, description of surgical technique but no reported strabismus outcome).– Outcomes from Botulinum Toxin (BT) injections.– Poster abstracts.– Review papers reporting no original data.– Editorial articles. ** Exclusions due to: – Strabismus surgery outcomes reported in a heterogeneous cohort and not possible to extract outcomes in those undergoing strabismus surgeries for psychosocial reasons only.– Insufficient evidence reported to be able to determine postoperative outcomes of strabismus surgery in those undergoing strabismus surgery for psychosocial reasons.– Cohort already reported in an earlier study.

The results of the literature search are shown in Figure 1. Sixty-nine papers were included in the literature review reporting the outcomes of strabismus surgery undertaken for psychosocial reasons (see Table 2).

**Table 2:** Displaying the evidence included in the literature review.


AUTHOR	STUDY PURPOSE	PATIENTS	OUTCOME CRITERIA	TIME POSTOPERATIVE OUTCOME JUDGED	STUDY DESIGN

(Adams et al. 2016)	Investigating psychological issues in patients before and after strabismus surgery	All strabismus patients (n = 220) Age 17–88 No diplopia (n = 96)	Clinical assessment of success, partial success or failure using criteria 1 = largest angle of deviation <12PD (for ET, XT and HT), <20PD HoT; 2 = no (or rare) diplopia or visual confusion in primary and reading position; and 3 = no prisms or Bangerter foil occlusion Success = 3/3 criteria met Partial success = 1 or 2/3 criteria met Failure = 0/3 criteria met Psychological questionnaires (QoL: Adult Strabismus quality of life questionnaire AS-20, Mood: Hospital Anxiety & Depression Scale (HADS), Appearance related social anxiety and social avoidance: The Derriford Appearance Scale (DAS24), Beliefs about strabismus: Revised Illness Perception Questionnaire (IPQ-R), Beliefs about strabismus surgery: Treatment Representations Inventory (TRI), Fear of negative evaluation: Fear of Negative Evaluation (FNE), Perceived visibility: 7-point Likert scale from 1 (not at all visible) to 7 (extremely visible), Importance of appearance: The Centre of Appearance Research Salience Scale (CARSAL), Perception of their appearance: The Centre of Appearance Research Valence Scale (CARVAL), Satisfaction with social support: Multidimensional Scale of Perceived Social Support (MSPSS), Expectations about the outcome of surgery: designed by psychology team (ESSQ) Reasons for having surgery: designed by psychology team (RSSQ), Satisfaction with surgery: designed by psychology team	3 months clinical 3 and 6 months psychological	Prospective

(Akbari et al. 2015)	Persian version of AS-20 pre and postoperatively	All types of strabismus N = 112 Age 15–43 years	AS-20 (Persian version) VFQ-25 (Persian version) Diplopia (yes/no) PCT <10PD and ≥10PD	3 months	Prospective

(Alam et al. 2014)	Investigating AS-20 outcomes in those considered surgical success	Concomitant manifest strabismus >15PD (preop) successfully aligned within 10PD orthotropia N = 30 Age 11–34 years	AS-20	6 weeks 3 months	Prospective

(Aletaha et al. 2016)	Comparison of surgical techniques	Horizontal strabismus N = 54 Age 2–50 years	PCT Number of reoperations	3 months	Prospective

(Alkharashi & Hunter 2017)	Comparison of surgical techniques	All rectus strengthening procedures (resection or plication) N = 72 Age 1–86 years	Success = distance PCT ≤10 PD horizontal deviation and ≤6 PD vertical deviation Reoperation rate Postoperative alignment drift (change from immediate postoperative measurement to final visit measurement)	6–12 weeks	Retrospective

(Al-Wadaani 2017)	Retrospective review of all strabismus surgery	All non-adjustable strabismus surgery N = 96 Age 16–61 years	Improvement in deviation postoperatively	6–47 months	Retrospective

(Ball et al. 1993)	Case series of unexpected stereopsis postoperatively	N = 8	BSV tests		Retrospective

(Bayramlar & Gunduz 2006)	Review of long term outcome of strabismus surgery in dense amblyopes (6/60 or worse)	N = 33 Age 8–61 years	Krimsky measurement of deviation Success ±12 PD deviation	2 months and 24–108 months	Retrospective

(Beauchamp et al. 2003)	Review of strabismus outcomes (all patients combined)	All patients who had strabismus surgery (6 centres) N = 299 Age 16 years +	Success alignment = ≤8 PD horizontal deviation and ≤2 PD vertical deviation Success motor = ≤ +1 overaction Success sensory = no diplopia	1 day–19 months	Multicentre retrospective

(Berland et al. 1998)	Patients undergoing 8–9mm bilateral LR recession for XT	N = 30	Abduction limitation Reoperation rate	3–30 months	Retrospective

(Biglan et al. 1994)	Comparison of surgical procedures	All strabismus patients (all aetiologies) N = 24 adjustable N = 113 nonadjustable Mean age 43 and 42	Success = ±8 PD horizontal deviation and ±4 PD vertical deviation % success BSV Correction of diplopia	1 week and 6 weeks	Retrospective

(Bucci et al. 2009)	Horizontal saccades and vergence pre and postoperatively	With and without BSV N = 9 Age 8–20 years	PCT BSV Saccades (measured onset: time to reach 5% of peak velocity, offset: time when velocity reduced to <10 degrees/sec, gain, mean velocity) Vergence (convergence and divergence) (measured onset: time when velocity reached > 5 degrees/sec, offset: time when velocity reduced to 5 degrees/sec, gain, mean velocity). Saccades combined with vergence	2 weeks–2 months and 3–10 months	Prospective

(Burke et al. 1997)	Psychosocial implications of strabismus and surgery	All had surgery for alignment N = 31 Age 18–68 years	Self-reporting repertory grid – self rating psychosocial issues (pre op and post op) PCT	3 months	Prospective

(Chang et al. 2017)	Binocular summation in strabismic amblyopia and effect of surgery	N = 15 strabismic amblyopia & Sx N = 30 normal N = 30 strabismus but no amblyopia	VA at 100%, 2.5% and 1.25% contrast (BEO & monocularly) Calculation of BiS Stereopsis PCT	6–10 weeks	Prospective

(Cifuentes et al. 2018)	Outcomes after 3 muscle surgery for large angle horizontal deviations	Consecutive patients having 3 muscle surgery for large angle horizontal strabismus patients N = 28 Age 1–79 years	Motor alignment success criteria: Dist = Primary position 10PD residual deviation – 4PD consecutive deviation and no induced lateral incomitance 5PD between lateral gazes Nr = Primary position 10PD residual deviation – 4PD consecutive deviation Sensory success: improvement in stereopsis of 2 octaves Overcorrection >4PD consecutive deviation Dist & Near (primary position) Undercorrection >10PD deviation Dist & Nr (primary position)	6 weeks–57 months	Retrospective

(Currie et al. 2003)	Outcomes after surgery for large angle XT	Consecutive patients having surgery for large angle XT N = 26 Age 14–68 years	PCT Success criteria Dist = ≤10 PD heterotropia or phoria BSV Subjective question – Happy? Yes/No	8–12 months 18–36 months	Retrospective

(Dadeya et al. 2002)	Use of a drug during surgery to reduce restrictions postoperatively	Strabismus patients having a second surgery, +ve FDT but ≤25 PD N = 20 Age 6–25 years	PCT FDT score Success criteria Satisfactory = ± 5 PD of orthophoria Undercorrection Overcorrection	1, 4 and 8 weeks then monthly for 12 months Outcome at 12 months	Prospective RCT

(Daga et al. 2022)	Comparison of surgical techniques in XT	N = 80 Intermittent or constant XT Mean age = 23 (range unclear) Two groups of different surgical techniques	PCT Exo drift Slit lamp assessment (ocular surface changes, muscle lump related changes)	1 day, 1 week, 1 month, 3 months and 6 months	Prospective randomised intervention

(Dawson et al. 2013)	Outcomes of strabismus treatment with poor VA (6/24 – PL)	Strabismus treatment outcomes in patients with reduced VA BT n = 11 (n = 2 then Sx) Sx (n = 8 total) N = 17 Age 19–74 years	PCT Comments documented in clinical notes about patient satisfaction postoperatively	2 weeks	Retrospective

(Dotan et al. 2014)	Strabismus surgery in patients with unilateral vision loss and horizontal strabismus	Horizontal strabismus and unilateral VA in worst eye 1.0 or worse, VA in better seeing eye 0.3 or better N = 21 Age 3–64 years	PCT Success ≤10 PD horizontal deviation and 1 surgical procedure was required Not success if >10PD or if >1 surgical procedure required	6–60 months	Retrospective

(Eino & Kraft 1997)	Adjustable surgery for horizontal deviation	Compared predetermined target angle (after adjustment) to deviation at 6–8 months N = 109 Age 15–72 years	PCT Drift from final alignment to 6–8 month measurement (in PCT and direction) Success if <10PD	Final alignment after adjustment 1–2 weeks 6–8 weeks 6–8 months	Retrospective

(Elkamshoushy & Langue 2019)	biLR recession for recurrent XT (prev biMR resect)	Previous biMR resection for XT, but recurrent XT N = 15 Age 20–31 years	PCT OM limitation of ABDuction Success 8PD ET – 10PD XT	6 months	Retrospective

(Estes et al. 2020)	Strabismus surgery, social anxiety and self consciousness	N = 95 >18 years old	Questionnaire to evaluate self-consciousness (private and public) and social anxiety (self-consciousness survey instrument) Pre-op and post-op	6 months	Prospective

(Faridi et al. 2007)	All surgery for primary XT, no previous surgery	Intermittent or constant XT N = 124 Mdn age at surgery 13 years (IQR 6–34 years)	Good motor outcome = ± 10PD orthotropia (SPCT) BSV	1–79 months	Retrospective

(Fatima et al. 2009)	Report postoperative BSV when none predicted preoperatively	Constant strabismus with no predicted BSV (free space with prisms) N = 15 Age 12–40 years	BSV Success = ≤10 PD horizontal deviation and ≤4 PD vertical deviation	6 weeks	Retrospective

(Felius et al. 2001)	Re-recession of MR for recurrent ET	N = 115 Age 11 months–77 years	PCT Success ET ≤10 PD or XT ≤8 PD OM on versions (underaction of MR)	4 weeks–8 months Long-term follow-up 8–120 months	Retrospective

(Frangouli & Adams 2013)	Amniotic membrane in complex repeat strabismus surgery	Strabismus surgery complicated by fibrosis, range of aetiology N = 8 Age 10–70 years	PCT Objective improvement Subjective improvement in patient symptoms (mainly relating to diplopia, but also includes report of binocular field of vision) Need for further interventions	9–24 months	Retrospective

(Ganesh et al. 2011)	Long-term follow-up of patients who had surgery for childhood ET	Surgery for ET until aligned to 0-10PD ET. Review 32–44 years later N = 85 Age 2–24 at surgery	Initial surgery success = 0–10PD ET Incidence of consecutive XT = ≥10PD XT Near and Dist Reoperations OM restriction of ADDuction BSV	32–44 years	Prospective long term follow up study

(Ghiasi et al. 2013)	Psychosocial improvement after strabismus surgery	N = 124 Age 15 years+ (71% no diplopia)	Used questionnaires from (Nelson et al, 2008) translated (Iranian population)	3 months	Prospective

(Gigante et al. 2018)	10-year follow-up after monocular surgery for large angle ET	Range of aetiologies of large angle ET N = 36 Age at surgery 4–58 years	PCT Good ≤15PD Fair 16–20PD Poor >20PD Rate of consecutive XT	6 months 10 years	Prospective long-term follow-up

(Glasman et al. 2013)	QoL following strabismus surgery – all patients with complete data	Horizontal and vertical deviations N = 86 Age 17–76 years	PCT AS-20 (total, function subscale and psychosocial subscale)	12 days–1 year	Prospective

(Gusek-Schneider & Boss 2010)	Secondary sensory strabismus surgery outcomes	All patients having surgery for secondary sensory strabismus N = 26 Age 3–45 years	PCT Dist VA BSV Diplopia yes/no Patient satisfaction with surgery yes/no	3 months Last follow up (1 year 8 m – 13 years 3 m)	Retrospective

(Hatt et al. 2010)	HRQoL questionnaires in strabismus surgery	All strabismus, with diplopia (n = 80) and without diplopia (n = 26) N = 106 Age 18–84 years	AS-20 VFQ-25 PCT (SPCT) Success criteria no diplopia/visual confusion in primary position or for reading <10PD heterotropia primary position Near or Dist No prism/Bangerter foil/occlusion No symptoms relating to misalignment or strabismus surgery Partial success No diplopia/visual confusion in primary position or reading <20PD heterotropia in primary position at Dist and Near No prism/Bangerter foil/occlusion Mild/intermittent symptoms relating misalignment or strabismus surgery (eyestrain/blur/photophobia/suture reaction) Failure Diplopia/visual confusion in primary position and reading ≥20PD heterotropia in primary position at Dist or Near Using prism/Bangerter foil/occlusion Moderate/severe symptoms related to misalignment or strabismus surgery	4–13 weeks	Prospective

(Hatt et al. 2018)	Identify factors associated with failure of AS-20 scores to improve following strabismus surgery	All strabismus patients – looked at failure to improve on each of the 4 AS-20 domains N = 276 Age 18–91 years	PCT (SPCT) Near 1/3m and Dist 3m AS-20 (4 domains) Diplopia questionnaire Center for Epidemiologic Studies Depression Scale–Revised (CESD-R) (depressive symptoms) Type-D Scale 14 questionnaire (type-Distressed [type-D] personality)	6 weeks	Prospective

(Hatt et al. 2012a)	Changes in HRQoL 1 year after successful strabismus Sx	All strabismus patients included, all aetiologies N = 73 Age 18–88 years	PCT (SPCT & PACT, but SPCT used in criteria) AS-20 Change in AS-20 psychosocial score Change in AS-20 function score Revised diplopia questionnaire Success: no/rare diplopia/visual confusion straight ahead at distance and for reading, <10PD heterotropia in primary position at distance and near Partial success: diplopia/visual confusion ‘sometimes’ or less straight ahead distance and for reading (with or without prism), and <15PD heterotropia Failure: either diplopia/visual confusion was ‘often’ or ‘always’ straight ahead distance or for reading, >15PD heterotropia at distance or near, or the patient was using a Bangerter foil/occlusion	6 weeks (but between 4–14 weeks) 1 year (but between 5–22 months)	Retrospective

(Hatt et al. 2016)	Incorporating HRQoL into the assessment of outcome after strabismus surgery	Assess ‘failures’ by motor and diplopia criteria and evaluate change in HRQoL. Any strabismus type with and without diplopia. All aetiologies. N = 227 Failures (n = 40) Age 18–88 years	PCT (SPCT) Dist 3m and Near 1/3m Diplopia questionnaire AS-20 Motor criteria Diplopia criteria Failure: if 1 of the following criteria was met: (1) SPCT ≥15 PD (horizontal or vertical) at distance or near; (2) diplopia or visual confusion was present more than “sometimes” straight ahead at distance or for reading (unless atypical diplopia due to decompensated childhood strabismus was present preoperatively, in which case diplopia was allowed postoperatively); (3) occlusive patch/Bangerter foil needed. Partial success: SPCT ≤15 PD (horizontal and vertical) at distance and near, and diplopia/visual confusion was present never/rarely/sometimes. Correction of diplopia with prism was allowed. Success: if SPCT <10 PD (horizontal and vertical) at distance and near, and diplopia/visual confusion was present never or only rarely.	1 year (but between 5 months – 2 years)	Prospective

(Hertle 1998)	Compare clinical characteristics of strabismus surgery with different onset	Compared strabismus onset before visual maturation (BVM) and after visual maturation (AVM). All surgery and all patients reported. N = 255 Age 14–72 years	PCT BSV Subjective report Success – sensory: restoration of function field of BSV (>20◦), regaining central or peripheral fusion, orthotropia or heterophoria in primary position and at near Success – motor: absence of binocular function without diplopia, horizontal alignment <12PD and vertical alignment <5PD in primary position and near Success – subjective: subjective interpretation on improved eye position, binocular function and appearance (including happy/unhappy with eye position, tolerant/intolerant of residual diplopia, happy/unhappy with eye movement) Incomitance = difference ≥8PD	6 months–5 years	Retrospective

(Jackson et al. 2006)	What are the psychosocial benefits of strabismus surgery	All strabismus patients. N = 46 Age 16–61 years (40% diplopia 60% no diplopia)	PCT 1/3m Visual Analogue Scales (VAS) (0–10) for 5 questions on coping, lifestyle, worry, noticeable strabismus, strabismus severity Derriford Appearance Scale (DAS-24) Hospital Anxiety and Depression Scale (HADS) WHOQoLBref (four quality of life domains: physical, psychological, social, and environmental) BSV	3 months (but between 1–6 months)	Prospective

(Ji et al. 2020)	Self-reported sense of deviation in adults successfully aligned with surgery	All deviations N = 91	PCT EOM BSV AS-20 (Chinese version) Self-report of deviation: no deviation/still have some deviation/still have obvious deviation (some and obvious were classed as self-reported sense of deviation) Success: (>1 year of follow up) no/rare diplopia/visual confusion in primary position and for reading, <10PD horizontal deviation, <5PD vertical deviation at near or dist	Follow up >1 year Last postoperative visit (12–42 months)	Retrospective

(Jung & Kim 2018)	Surgical outcomes in sensory XT	Unilateral visual loss and constant horizontal strabismus VA <6/30 (0.7) N = 64 Age 18–71 years	Success = <10PD dist Failure = recurrence or overcorrection Recurrence ≥10PD XT Overcorrection ≥10PD ET	1 year	Retrospective

(Kannam et al. 2021)	Surgical outcomes of horizontal rectus muscle transplantation in recurrent and residual strabismus	Case series N = 7 Age 16–40 years (N = 6 XT, N = 1 ET)	PCT Good alignment (cosmetic success) <12PD Duction limitation	1 week, 6 weeks and final visit (3–6 months)	Retrospective

(Kattan et al. 2016)	Binocular summation and stereoacuity after strabismus surgery	All types of strabismus and surgery N = 130 Age 20–60 years	VA 100% contrast VA reduced contrast 2.5%, 1.25% in dimly lit room Binocular summation Stereoacuity near and dist Diplopia Measures only taken postoperatively	2 months	Prospective case series

(Keskinbora et al. 2011)	Long standing infantile ET – outcomes in late surgery	Alignment and BSV despite late surgery and early onset ET N = 21 Age 8–26 years	PCT BSV <5PD heterotropia = orthotropia Residual ET ≥5PD ET Exotropia ≥5PD XT	3–9 years	Retrospective

(Kim et al. 2008)	Reoperation in sensory strabismus	N = 11 Age 4–33 years	PCT Success = 0–10PD	1 month Last visit (1–48 months)	Retrospective

(Kim et al. 2016)	Self-identity in strabismus and after surgery	N = 351 Age 19 years + 3 groups Strabismus (n = 96) Surgery age 4–15 years (n = 108) No strabismus (n = 147)	Korean self-identity scale (subscales: subjectivity, self-acceptance, future confidence, goal orientation, initiative, and familiarity)	3 independent groups – not before and after surgery	Retrospective

(Kishimoto & Ohtsuki 2012)	VF14 in different ophthalmic conditions	Concomitant and incomitant strabismus N = 625 Age 40–85 years	VF-14 questionnaire PCT BSV (Concomitant group)	3 months	Prospective

(Koc et al. 2013)	Strabismus surgery outcomes – does binocular vision make a difference to QoL	N = 61 Age ≥18 years	AS-20 A&SQ (Amblyopia and strabismus questionnaire) BSV Diplopia score (from A&SQ) Motor success <10PD horizontal deviation and <5PD vertical deviation Sensory results BVP (binocular vision positive) and BVN (binocular vision negative)	3 months	Prospective

(Kushner 1994)	Visual field (binocular or BEO) after surgery for ET	ET Sx N = 37 Age 16–62 years	PCT Binocular VF (BEO) BSV (BG)	6 weeks	Prospective

(Kutschke & Scott 2004)	PAT in ET (childhood onset, but Sx when visually mature)	All types of ET N = 85 Age 9–70 years	Success 0–8PD SPCT at near and dist + peripheral fusion Those with no BSV postoperatively are reported	6 weeks to 13.7 years	Retrospective

(Lee et al. 2013)	Postoperative change in spatial localisation after XT surgery	XT N = 60 Age 4–43 years	PCT Computer touch screen – spatial localisation (pointing errors)	1 day 1 month	Prospective

(Liebermann et al. 2013)	Compare long-term outcomes in reoperation of horizontal strabismus-adjustment vs. no adjustment following surgery	ET and XT With and without potential BSV N = 89 Age 12–83 years	Success: <10PD dist deviation (primary and near), no/rare diplopia (primary and reading), no prism or occlusion Partial success: ≤15PD dist deviation (primary and near) without prism, diplopia none/rare/sometimes in primary and reading, prism allowed, no occlusion Failure: if any of these are met >15PD dist deviation in primary or reading, diplopia always/often in primary and reading, needs occlusion	6 weeks (but 3–21 weeks) 1 year (but 23 weeks–2 years)	Retrospective

(Liebermann et al. 2014)	Improvement in specific function HRQoL concerns after strabismus surgery in nondiplopic adults	N = 20 Age 22–79 years	Same success criteria as Liebermann et al. (2013) AS-20 PCT BSV	1 year (but 6–19 months)	Retrospective

(Lipton & Willshaw 1995)	Comparison of surgery accuracy – specialist centre compared to general	N = 205 Age ?	PCT Success: Grade 1 within 0–5 PD of surgical goal Grade 2 within 6–10 PD of surgical goal Grade 3 >10PD of surgical goal	6 months	Prospective multicentre study

(McBain et al. 2014b)	QoL and mood postoperatively	Range of aetiologies N = 210 Age 17–88 years	PCT (APCT 6m) Self-reports of pain, swelling, scarring, redness 0–10 scale At 3 months: Success: 3 out of 3 criteria met: <12PD ET/ XT/HT <20PD HoT, no/rare diplopia/visual confusion in primary position and reading, no prism/occlusion needed Partial success: 1 of the 3 criteria met Failure: 0 out of 3 criteria met AS-20 Success AS-20: >17.7-point increase in psychosocial subscale and >19.5-point increase in function subscale (>95% LOA) Psychosocial measures: Revised Illness Perception Questionnaire (IPQ-R) Treatment Representations Inventory (TRI) Fear of Negative Evaluation (FNE) scale The Derriford Appearance Scale (DAS24) Perceived Visibility of Strabismus Salience of Appearance scale (CARSAL) Valence of Appearance scale (CARVAL) Multidimensional Scale of Perceived Social Support (MSPSS) Hospital Anxiety and Depression Scale (HADS) Questionnaires: Reasons for strabismus surgery (RSSQ) Expectations of strabismus surgery (ESSQ) Additional questions: Do you regret having strabismus surgery: Yes definitely 1 – Not at all 4 Would you go through the surgery again: No hesitation at all 1 – Certainly not 4	3 months 6 months	Prospective

(Menon et al. 2002)	Psychosocial aspects of strabismus	All having surgery for alignment N = 40 Age 15–25 years	Semi-structured interview to complete questionnaire and score questionnaire items (pre-op and post-op) Neuroticism questionnaire	3 months	Prospective

(Murray et al. 2007)	Changes in binocular status after late surgery for infantile ET	N = 17 (if aligned 0–8PD at 1 day post op)	BSV (Worth 4 dot test, BG, Titmus, fusion on Synoptophore) Visual field BEO	Last follow-up N = 6 < 1 month N = 5 < 3 months N = 6 > 1 year	Retrospective

(Natung et al. 2022)	Evaluation of surgical dose calculation for horizontal, concomitant strabismus	N = 38 Age 18–47 years N = 19 sensory strabismus	Measurement of deviation only (PCT or Krimsky) Compared correction achieved to correction expected from surgical dose	3 months	Retrospective

(Nelson et al. 2008)	Psychosocial impact of strabismus and surgery	N = 128 Age ≥ 15 years N = 20 teenagers N = 108 adults	Postoperative telephone interviews to complete questionnaire about psychosocial issues (1–10) and postoperative outcome (1–7)	Unclear	Retrospective

(Ozates et al. 2019)	Psychological impact of strabismus surgery	N = 83 Age 14–21 years XT & X(T)	Grouped by constant/manifest deviation XT or X(T) Turkish versions of: Social Appearance Anxiety Scale (SAAS) Depression subscale of the HADS (HAD-D Brief Fear of Negative Evaluation Scale (BFNE) state anxiety subscale of State-Trait Anxiety Inventory (STAI-S) trait anxiety subscale of State-Trait Anxiety Inventory (STAI-T)	1 year	Prospective

(Pineles et al. 2015)	Binocular summation after strabismus surgery	All strabismus types N = 97 Age 2.5–90 years	VA high contrast (100%) VA low contrast (2.5% and 1.25%) Binocular summation calculation PCT Diplopia Success = 0–10PD horizontal strabismus and 0–4PD vertical strabismus	6–10 weeks	Prospective

(Ribeiro et al. 2014)	QoL in strabismus	N = 101 Age 7–67 years 75% no surgery 25% had surgery	Semi-structured interviews to complete questionnaire (own modified version of AS-20)	?	Prospective

(Sandercoe et al. 2014)	Retrospective review of strabismus surgery	Categorised reasons for surgery (78% for psychosocial reasons) N = 83 Mean age 37 years	PCT BSV Diplopia Objective criteria for success <10PD and acceptable 10–20PD results Subjective criteria = satisfaction with surgical outcome (very satisfied/satisfied/neutral/unsatisfied/very dissatisfied)	Mean 16 weeks	Retrospective

(Sefi-Yurdakul et al. 2022)	Comparison of surgical techniques for consecutive XT	N = 49 Age 5–50 years Four groups of different surgical procedures compared	PCT Success <10PD	Last follow up visit (7–17 months)	Retrospective

(Sim et al. 2018)	Factors associated with patient perception of success	N = 87 Age 16–83 years 35% had no diplopia	AS-20 (used >95% limits of agreement as evidence of change) Diplopia PCT	24–126 days	unclear

(Tibrewal et al. 2021)	Surgical outcomes of primary EOM transplantation for large angle XT	N = 10 Large angle XT ≥60PD Age 2–30 years	Measurement (PCT or modified Krimsky test) Motor success ≤10PD Restorative success ≤12PD ABDuction limitation	1 week, 6 weeks, final visit (8.6–38.8 months)	Retrospective

(Wang & Nelson 2011)	Sm-mod ET surgery outcomes	N = 123 Age 11 months–48 years	Success 0–5PD (PCT near and dist, primary position and lateral gaze)	6 months Last follow-up (6 months–8 years)	Retrospective

(Wortham & Greenwald 1989)	Binocular visual field in ET	N = 10 Age 22–49 years	PCT Visual field BEO BSV	1–2 months	Retrospective

(Xu et al. 2012)	Psychosocial effect of strabismus surgery	N = 56 Age 16–49 years No diplopia pre-op 64% surgery for BSV 36% had surgery for alignment	Own questionnaire (social function and psychological function scores) CT = fair alignment (small manifest deviation) or excellent alignment (no manifest deviation)	2–3 months	Prospective

(Xu et al. 2016)	Long-term follow-up and HRQoL following strabismus surgery	N = 122 Compared AS-20 results to control group without strabismus N = 89	AS-20 (Chinese version) PCT OM BSV Sense of deviation (no deviation/still have some deviation/still have obvious deviation) Diplopia	Last follow up 12–24 months)	Prospective


BEO both eyes open, BiS binocular summation, BSV binocular single vision, CT cover test, E esophoria, ET esotropia, HRQoL health related quality of life, OM ocular movements, PCT prism cover test, PD prism dioptres, VA visual acuity, X exophoria, XT exotropia.

## Discussion

Evidence of surgical outcomes in adults undergoing strabismus surgery for psychosocial reasons was needed to improve our understanding of the potential risks and benefits of undergoing strabismus surgery. Postoperative outcomes from strabismus surgery were typically reported within a heterogeneous cohort.

### Delphi study and core outcome sets

A Delphi study attempted to identify areas of consensus and disagreement amongst Ophthalmologists when defining success following strabismus surgery (Serafino et al. 2019). A range of different strabismus types and aetiologies were included, however some of the questions included in the Delphi study were pertinent to adults with strabismus and psychosocial symptoms. There was a lack of consensus reached on the time point at which postoperative outcomes should be evaluated, the deviation size considered successful postoperatively and how the deviation should be measured (simultaneous PCT, alternate PCT or both). Consensus was reached in support of some strabismus conditions having unique outcome criteria (for example, sixth cranial nerve palsy) and for BSV outcomes to be included in the definition of success for some strabismus types. Al Jabri et al. (2019) also highlighted the difficulty in comparing studies reporting strabismus outcomes due to a lack of ‘core outcome measures’ used. The COMET Initiative (‘COMET Initiative: Core Outcome Measures in Effectiveness Trials’) aims to encourage core outcome set development and use in clinical trials. A core outcome set is the minimum set of measurements that should be taken and reported in a clinical trial of a specific condition. Core outcome sets are therefore useful as they allow comparison of study results and outcomes across different studies. Al Jabri et al. (2019) identified the outcome measurements most commonly used and reported in amblyopia, strabismus, and ocular motility disorder studies, as well as highlighting that consensus was required to develop core outcome sets for trials and research into these conditions. Of note in the strabismus studies were the most commonly reported core outcome measurements of a near and distance measurement of the deviation, binocularity, HRQoL, and adverse events, with some studies additionally reporting visual acuity (VA) and control of the deviation.

### Eye alignment

Overwhelmingly the most commonly reported strabismus surgery outcome was the primary position angle of deviation, usually in the distance, measured by the prism cover test (PCT) and reported in prism dioptres (PD). Additionally, stating criteria for ‘success’ based on the strabismus size postoperatively was common. These had the advantage of allowing comparison between the percentage successfully aligned with surgery, even when different procedures or techniques were compared. Typically, a target angle considered surgical ‘success’ was stated and a success rate or percentage achieving success postoperatively was reported. A successful angle was often 0–10PD horizontal deviation (Alkharashi & Hunter 2017), with some specifying 0–5PD (Wang & Nelson 2011), 0–8PD (Beauchamp et al. 2003), or 0–15PD (Gigante et al. 2018). Vertical angles considered successful were 0–2PD (Beauchamp et al. 2003), 0–4PD (Biglan et al. 1994), 0–5PD, 0–6PD (Alkharashi & Hunter 2017), although vertical deviations as large as 12PD hypertropia (HT) and 20PD hypotropia (HoT) were also considered successful (Adams et al. 2016).

Additional factors could be included in the definition of success. For example, a large prospective multicentre study compared outcomes between different centres (specialist or general) and success was graded based on the preoperative surgical aim. Postoperatively success was graded as within 0–5PD (grade 1 success), 6–10PD (grade 2) or greater than 10PD (grade 3) compared to the surgical goal (Lipton & Willshaw 1995). The original angle of deviation may be included, for example Cifuentes et al. (2018) reported success criteria of residual deviation up to 10PD and consecutive deviation up to 4PD, with no induced lateral incomitance after surgery for large angle horizontal strabismus. A difference in the esotropia (ET) and exotropia (XT) angle, depending on the strabismus type or aim of the procedure may also be specified. For example, in a large retrospective study reporting re-recessions for recurrent ET, success was considered to be 0–10PD residual ET or 0–8PD consecutive XT (Felius et al. 2001). Outcome measures relating to the specific surgical procedure may also be included. For example, the amount of abduction limitation was an outcome of bilateral lateral rectus (LR) recessions for recurrent XT (Elkamshoushy & Langue 2019) and incidence of consecutive XT and reoperation rate were outcomes in a long-term follow-up of surgery for childhood-onset ET. Postoperative drift (Alkharashi & Hunter 2017; Eino & Kraft 1997), whether reoperation was required (Aletaha et al. 2016; Alkharashi & Hunter 2017) and complications (Faridi et al. 2007) have also been reported as outcome measures, with some including need for reoperation as failure (Dotan et al. 2014).

### Diplopia and BSV

Surgical procedures for planned visual benefit typically included the aim of surgery as an outcome, for example the percentage achieving BSV or improvement in BSV postoperatively (Cifuentes et al. 2018). Surgery for strabismus and psychosocial symptoms would not typically include visual symptoms as outcomes, unless postoperative BSV (Ball et al. 1993) or diplopia occurred. Gusek-Schneider and Boss (2010) included diplopia (yes/no), PCT, VA, BSV, and patient satisfaction (yes/no) when reporting postoperative outcomes in secondary sensory strabismus (n = 26). The challenge of different outcome measures for different patients was recognised in a retrospective study that grouped patients by strabismus onset, before or after visual maturation (n = 255) (Hertle 1998). Success criteria were divided into sensory and motor success. Sensory success included restoration of BSV or functional field of BSV. Motor success included orthotropia or heterophoria in primary position and at near. In the absence of BSV and diplopia, motor success included alignment, with a less than 12PD horizontal and less than 5PD vertical deviation considered successful.

### Defining success

Increasingly a range of factors have been included in a definition of success to reflect the view that eye alignment is not the only important outcome measure. Hatt et al. (2010) reported success, partial success, and failure outcomes, although their cohort included patients both with diplopia and BSV, and without. Success included no diplopia or visual confusion in primary position or when reading, less than 10PD heterotropia in primary position at both near and distance, no prism or occlusion, and no symptoms relating to strabismus or strabismus surgery. Partial success included the same criteria, but with a less than 20PD deviation and mild or intermittent symptoms (relating to the strabismus or surgery). Failure included diplopia or visual confusion in primary position and when reading, 20PD heterotropia or larger, using prism or occlusion, and moderate or severe symptoms (relating to strabismus or surgery). Their criteria were later refined to include success as having no or rare diplopia, partial success as less than 15PD with diplopia sometimes, with and without a prism, and failure as greater than 15PD heterotropia and diplopia often or always at distance or reading (Hatt et al. 2012a, 2016; Liebermann et al. 2013, 2014). In a large prospective cohort study (n = 210), patients with all types of strabismus were recruited to a study investigating QoL and mood, before and after strabismus surgery. Deviation size less than 12PD ET, XT, or HT and less than 20PD HoT, no or rare diplopia in primary position and reading, and no prism or occlusion needed were used as the criteria determining success, partial success, and failure. Success required all three criteria, partial success required one of three criteria and failure required none of the criteria were met (Adams et al. 2016; McBain et al. 2016).

### Patient perception of the postoperative outcome

Success from the patient’s perspective may be different to the clinician’s perspective. In recognition of this, some studies included objective and subjective outcomes postoperatively (Frangouli & Adams 2013) or asked patients to report their eye alignment, binocular function, and appearance subjectively (happy/unhappy) (Hertle 1998). In a retrospective study (n = 83) 78% underwent surgery for psychosocial reasons (without diplopia) and both objective and subjective success criteria were used to report the outcomes. Eighty-three percent of all patients had a successful outcome, both objectively (deviation less than 10PD) and subjectively (very satisfied) (Sandercoe et al. 2014).

### Questionnaires

Increasingly, questionnaires for patients to self-report visual function, QoL, HRQoL, and patient reported outcome measures (PROMs), both generic and those developed specifically for strabismus, have been used pre- and postoperatively (Hatt et al. 2016). Using telephone interviews to complete questionnaires postoperatively (n = 128), patients reported satisfactory eye position (98%) and improved self-esteem (85%), abilities to meet new people (65%), interpersonal relationships (27%), and abilities to try new activities (16%). Younger patients reported greater improvements postoperatively and a larger preoperative deviation was associated with greater improvements in self-esteem and self-image postoperatively (Nelson et al. 2008). Interviews have been used to complete questionnaires rather than explore patient perceptions of postoperative outcome (Menon et al. 2002; Ribeiro et al. 2014). Menon et al. (2002) reported 97.5% of their cohort (n = 40) had improved appearance, relationships with others, self-esteem, and self-confidence postoperatively. Postoperatively 37.5% changed future plans, and 95% reported trying new activities or things that had previously been avoided.

Ghiasi et al. (2013) used a similar questionnaire to Nelson et al. (2008) to prospectively evaluate changes three months after strabismus surgery. All aspects of the questionnaire were reported as improved postoperatively. A high percentage of patients reported improved self-esteem (89%), improved relationships (82%), being able to meet new people (79%), and being better at their job or work (76%) postoperatively. A smaller percentage of patients also reported having improved chances of employment (53%) and being able to try new activities (36%) postoperatively. Gender and direction of strabismus did not affect the results.

Burke et al. (1997) asked patients (n = 31) seeking surgery for alignment only to complete questions about psychosocial issues, rating themselves on a five-point scale preoperatively and three months postoperatively. Patients reported significantly improved psychosocial functioning postoperatively. However, they also reported less than ‘ideal world’ results and that others would rate them less highly than they rated themselves postoperatively. Age did not affect the results, but females and ETs reported greater improvements in psychosocial functioning compared to males and XTs. Greater improvements in HRQoL in females postoperatively has also been reported using the AS-20 (Akbari et al. 2015; Alam et al. 2014; Glasman et al. 2013).

Xu et al. (2012) used their own questionnaire to investigate social and psychological effects of strabismus and surgical correction. None of the cohort (n = 56) had diplopia preoperatively and 36% had surgery for alignment only (psychosocial reasons). The most common postoperative outcomes (and the percentage of respondents reporting that outcome) were change in appearance (96%), change in self-esteem or self-confidence (96%), change in relationships with friends (91%), trying activities previously avoided (82%), and changing plans for the future (68%). However, it is unclear which outcomes were gained by those having surgery for alignment only.

### Visual function

The VFQ-25 questionnaire is used to measure self-reported visual function and the AS-20 questionnaire is reported to measure HRQoL. Visual functioning questionnaires have measured improved visual function after strabismus surgery (VF-14) (Kishimoto & Ohtsuki 2012). The VFQ-25 was compared to the AS-20 in a prospective study (n = 106). In those without diplopia (n = 26), the AS-20 was better able to discriminate between surgical success (total or partial) and failure than the VFQ-25; however, VFQ-25 scores did improve. In those without diplopia, successful outcomes had significantly higher VFQ-25 scores (composite score, all vision-specific subscales, driving subscale, and colour vision subscale) (Hatt et al. 2010). Akbari et al. (2015) reported good correlation between the AS-20 and VFQ-25 (Persian versions) but did not analyse their results based on surgical success. Jackson et al. (2006) used visual analogue scales to report coping, lifestyle, worry, noticeable strabismus, and strabismus severity on a 0–10 scale, as well as the DAS-24, HADS and the WHOQoLBref. Strabismus surgery (n = 46) resulted in significant improvements in QoL, psychological and physical functioning, which were greater in those without diplopia.

### AS-20

The AS-20 (Hatt et al. 2009) has become the most commonly used HRQoL questionnaire in strabismus (Adams et al. 2016; Alam et al. 2014; Glasman et al. 2013; Hatt et al. 2010; Hatt et al. 2012a, 2016; Hatt et al. 2018; Ji et al. 2020; Koc et al. 2013; Liebermann et al. 2014; McBain et al. 2016; Sim et al. 2018). Despite not being specific to strabismus with psychosocial symptoms, surgery in these patients has improved both psychosocial and functional aspects of the AS-20 (Alam et al. 2014; Hatt et al. 2010; Hatt et al. 2012a; Koc et al. 2013). Liebermann et al. (2014) reported all AS-20 functional elements improved postoperatively in patients without diplopia (n = 20), with the greatest improvements in stress, worry, needing to take breaks, enjoying hobbies, depth perception, and eye strain items. Significant improvements in self-reported visual function after strabismus surgery for psychosocial reasons were difficult to explain, as no visual change was measured using standard clinical vision tests. However, it is possible that a change in binocular field of vision may have occurred as this was not tested (Kushner 1994; Wortham & Greenwald 1989).

The AS-20 and A&SQ were used in a prospective study of adult strabismus surgery outcomes (n = 61) (Koc et al. 2013). Both questionnaires measured significant improvements in HRQoL three months postoperatively. Those with BSV postoperatively had significantly greater improvements in HRQoL scores on the functional subscales than those without BSV, but only when amblyopes were removed from the analysis. The change in overall scores and psychosocial scores (using both questionnaires) were not significantly different between those with and without BSV, highlighting that visual benefit postoperatively was not required for improvement in HRQoL.

Alam et al. (2014) used the AS-20 in a cohort of older children and adults undergoing first strabismus surgery (n = 30). None had diplopia, but it is unclear whether any had BSV. Significant improvements in AS-20 HRQoL were measured six weeks and three months postoperatively, with a greater improvement in females. Glasman et al. (2013) reported larger improvements in HRQoL (AS-20) in females, those with larger changes of the deviation and those with smaller strabismus postoperatively. Their prospective study of 17- to 76-year-olds (n = 86) found surgery led to improvements in all aspects of the AS-20; however, BSV and diplopia were not reported. Their cohort may therefore have included some surgery for visual benefit.

Adams et al. (2016) used the AS-20, as well a large battery of QoL and psychosocial measures in a prospective study of patients aged 17–88 years (n = 210). A range of aetiologies of strabismus were included and it is unclear how many had surgery for psychosocial reasons; however, 44% had no diplopia. Postoperatively there was a reduction in the number of patients reporting poor AS-20 HRQoL, from 85% to 68% at three and six months postoperatively. Other measures of social anxiety and avoidance, clinical anxiety, and depression also improved significantly. In a study reporting the same cohort (n = 210) McBain et al. (2016) used the AS-20 as the primary outcome measure. Strabismus surgery resulted in significantly improved HRQoL three months postoperatively, with no further improvements at six months. Improvements in HRQoL were not associated with clinical judgements of success, highlighting that clinical definitions of success may not adequately capture the postoperative result from the patient’s perspective. Postoperatively there were improvements in a wide range of psychosocial domains, as well as all aspects of the AS-20 (McBain et al. 2016). Using a questionnaire to evaluate self-consciousness, Estes et al. (2020) reported improved public (but not private) self-consciousness and improved social anxiety six months postoperatively. It was unclear how many of their cohort (n = 95) had surgery for psychosocial reasons, as some had diplopia (66%) and depth perception (62%) preoperatively. Using a range of psychological measures Ozates et al. (2019) demonstrated that constant XT (and no BSV) was significantly worse than intermittent XT and BSV. Surgery resulted in significant improvements in all psychological measures for the constant XT group, to the extent that there was no difference between constant and intermittent XT postoperatively.

Patients without diplopia reported significantly lower AS-20 psychosocial subscale scores preoperatively compared to those with diplopia. Interestingly, AS-20 function subscale scores were not significantly different. Postoperatively psychosocial and function subscale scores improved in all patients. Although the improvement in psychosocial subscale score was higher in those without diplopia, they continued to report lower postoperative psychosocial subscale scores than those with diplopia initially. The only factor predictive of a greater improvement in AS-20 HRQoL was socioeconomic status. Those from a more deprived area had a higher rate of success postoperatively (Sim et al. 2018)

Hatt et al. (2010) reported strabismus patients without diplopia gained significant improvements in AS-20 HRQoL, particularly if they had a successful result. Even those with ‘failure’ postoperatively reported AS-20 improvements, leading Hatt et al. (2016) to suggest success should include HRQoL improvements (beyond test-retest variability), in addition to improved alignment and diplopia. Having a distressed personality type, worse diplopia or depressive symptoms postoperatively, and coexisting facial abnormalities were associated with postoperatively failure, using the AS-20 as the outcome measure (Hatt et al. 2018). These results highlight that mental health as well as clinical factors influence the outcomes from strabismus surgery, a view shared by others (Adams et al. 2016; McBain et al. 2016). Hatt et al. (2012a) retrospectively reported outcomes in adults between 5–22 months postoperatively (n = 73), described as one-year results. Those who continued to meet success criteria (less than 10PD alignment and no or rare symptoms) maintained improved AS-20 results at one year compared to six weeks postoperatively. From six weeks to one year, those without diplopia showed stable function subscale results and further improved psychosocial subscale results. Ji et al. (2020) used the AS-20 (Chinese version) to investigate successful outcomes one year postoperatively, using similar success criteria to Hatt et al. (2016). Patients with BSV and diplopia were included. Despite successful strabismus surgery, 24% of their cohort (n = 91) still reported they had strabismus. Those who perceived a deviation postoperatively reported lower AS-20 scores and were more likely to have a larger vertical deviation (Ji et al. 2020).

Whilst motor outcomes (strabismus size) may be more likely to define surgery as successful, the method of AS-20 analysis has been shown to affect the results (Leske et al. 2010). Change in either AS-20 subscale, greater than 95% limits of agreement, was considered difficult to achieve (Hatt et al. 2012b), but relying on motor outcomes only may fail to capture improved symptoms or HRQoL (Hatt et al., 2016). The AS-20 was considered to have excellent test-retest variability and a low chance of a ceiling effect. A change in overall score of 14, psychosocial subscale score of 17.7, and function subscale of 19.5 were considered evidence of real change. Whilst different results were provided for those with and without diplopia (Leske et al. 2010), it was unclear whether the ‘without diplopia’ subgroup was strabismus with psychosocial symptoms only, as it may have included strabismus with BSV. A later evaluation of the AS-20 using Rasch analysis suggested refining the questions, the response options and the subscales to increase responsiveness to change in QoL. This resulted in removal of two questions from the previous function subscale (Leske et al. 2012).

Surgery has been reported to improve and normalise symptoms of anxiety and depression, HRQoL, daily functioning, and psychological adjustment postoperatively (Jackson et al. 2006); however, others report improved but not normalised HRQoL (Xu et al. 2016). Patients who perceived they had no strabismus postoperatively achieved greater HRQoL improvement (Xu et al. 2016). Kim et al. (2016) used a self-identity questionnaire to evaluate young adult males at a military service examination. Having strabismus negatively affected self-identity compared to those with no strabismus and those who had previously undergone strabismus surgery in childhood. There was no difference in self-identity between those who had previous strabismus surgery and those without strabismus. With the recognition that QoL is an important outcome from strabismus surgery, focus has shifted to consider whether psychosocial interventions preoperatively could improve QoL and psychosocial outcomes. No trials have yet been undertaken and this is an area for future research (MacKenzie et al. 2016).

### Timing of postoperative outcome

Clinical care of patients following strabismus surgery varied among different clinicians, hospitals, healthcare systems, and countries. Patients may be discharged at a specific time point if they are asymptomatic and happy with the surgical result, yet others may be kept under longer review. Strabismus surgery outcomes were reported at one week (Berland et al. 1998), two weeks (Dawson et al. 2013), one month (Kim et al. 2008), six weeks (Alam et al. 2014; Fatima et al. 2009), three months (Adams et al. 2016; Alam et al. 2014), six months (Adams et al. 2016; Lipton & Willshaw 1995), one year (Currie et al. 2003; Dadeya et al. 2002; Jung & Kim 2018) and later than one year (Currie et al. 2003; Felius et al. 2001). In some studies, the time at which outcome is being reported was unclear (Nelson et al. 2008). Reporting one-year postoperative outcomes had the advantage of providing longer-term data, yet many patients had been discharged and less data available for analysis (Liebermann et al. 2013). Longer-term postoperative outcomes may therefore be biassed and include a greater proportion of poorer outcomes that have not been discharged.

The last available follow-up (Al-Wadaani 2017; Beauchamp et al. 2003; Berland et al. 1998; Faridi et al. 2007) was commonly used to report postoperative outcomes, but this was also variable. Kim et al. (2008) reported postoperative outcomes following reoperation for sensory strabismus one month postoperatively and at the final postoperative visit, which ranged from 1–48 months. In contrast, the last available follow up visit ranged from six weeks to 13 years in a study of later surgery for childhood onset ET (Kutschke & Scott, 2004). Specific longer-term studies reporting outcomes after more than one year were less common but offered a unique view of postoperative stability and change over time. For example, 2–9-year follow-up (Bayramlar & Gunduz 2006), 3–9-year follow-up (Keskinbora et al. 2011) and 10-year follow-up (Gigante et al. 2018) have been reported. A unique Swedish prospective study invited adults who had surgery for childhood ET for review and reported 32–44-year follow-ups (Ganesh et al. 2011).

On balance, evaluation of strabismus surgery outcomes at, or later than, three months represented a useful and achievable time point, unless measuring longer term outcomes was the specific aim. For most patients this was thought to allow sufficient time for healing (Escardó-Paton & Harrad 2009), for eye alignment to stabilise and for the patient to adapt to their eye position. Measuring QoL outcomes at six months postoperatively was not significantly different to three months (McBain et al. 2016).

### Additional outcomes from strabismus surgery

Patients undergoing strabismus surgery for psychosocial reasons may achieve more than just psychosocial benefit, as shown by QoL or HRQoL improvements. Observational studies reporting additional postoperative changes are discussed in detail below.

#### Visual field

Patients have gained an enlarged peripheral visual field following surgery to reduce ET (Kushner 1994; Murray et al. 2007; Wortham & Greenwald 1989). Wortham and Greenwald (1989) reported ten patients with ET who postoperatively gained peripheral visual field, gaining a mean 16 degrees horizontally (range 5–30 degrees). Visual field size was measured using the Goldmann perimeter, I4e target. The gain in peripheral visual field was ipsilateral to the strabismic eye and occurred even in the presence of amblyopia (n = 3). Three patients gained some stereopsis postoperatively (range 80” of arc to Titmus fly). This suggested in patients without BSV the suppressed eye contributed to the peripheral field of vision. It also suggested that aligning the strabismic changed the amount, or extent, it contributed to the peripheral visual field. Anecdotally four patients reported visual improvement; however, patients were not asked to subjectively report visual change postoperatively. No follow up data were presented and comparisons with other patients were lacking. Murray et al. (2007) reported older children and adults (n = 17) with untreated infantile ET gained an expanded field of binocular vision (mean 32 degrees) postoperatively. However, in contrast to Wortham and Greenwald (1989), sensory fusion was always achieved in addition to binocular field expansion (Murray et al. 2007). Kushner (1994) reported that 34 of 35 patients (age 16–62 years) gained an expanded field of binocular vision postoperatively. The patient that did not gain field of binocular vision (n = 1) had unilateral poor vision and retinal abnormalities secondary to uveitis, which may have affected the postoperative outcome. Of those who gained field of binocular vision (n = 34), 29 had sensory fusion and 5 had suppression postoperatively (Bagolini glasses). Most patients that gained field of vision postoperatively were aware they had improved peripheral vision.

#### Unexpected binocular vision

Despite surgery for planned psychosocial benefit, unexpected BSV may occur postoperatively. For example, patients with longstanding large angle strabismus (n = 8) have achieved good stereopsis, mean 45” of arc (Titmus) (Ball et al. 1993). Eight patients (out of 20) achieved 60–400” of arc (Frisby Near Stereotest (FNS)) or 40–80” of arc (Frisby Davis distance stereotest (FD2)) one year postoperatively (Liebermann et al. 2014). Detailed reports of pre- and postoperative investigations of BSV in patients with strabismus are lacking. Retrospective studies aiming to identify factors that predict BSV postoperatively can lack complete outcome data (Umazume et al. 1997), leading to difficulty providing data on the proportion of patients who may achieve unexpected BSV or factors that may predict BSV postoperatively. These factors highlight the importance of assessing potential BSV preoperatively (Ball et al. 1993) and BSV outcomes postoperatively, even when it is assumed no BSV is possible (Murray et al. 2007).

#### Binocular summation

Strabismus surgery has been reported to improve binocular summation, with a greater effect measured using lower contrast (1.25%) acuity charts. This improvement can mean binocular summation is measured postoperatively, despite binocular inhibition preoperatively. Successful surgical alignment and later onset strabismus have both been associated with greater improvements in binocular summation postoperatively (Pineles et al. 2015). Yet, other studies have shown highly variable changes in binocular summation following strabismus surgery (Chang et al. 2017). Interpreting postoperative binocular summation data only, rather than change as a result of surgery, and interpretation of binocular summation data in isolation, rather than as part of an investigation of pre and postoperative BSV may also be misleading. Further evidence is required to establish whether binocular summation improves following all strabismus surgery, or whether BSV and stereopsis (pre- and postoperatively) affect the binocular summation outcome (Kattan et al. 2016).

#### Task performance

Patients have reported improved ability to perform daily activities (Nelson et al. 2008) and being able to work better (Ghiasi et al. 2013) when completing questionnaires postoperatively. Improved AS-20 function subscale results have been measured postoperatively even though patients have undergone surgery specifically for psychosocial symptoms or had no measurable visual change postoperatively (Alam et al. 2014; Hatt et al. 2010; Hatt et al. 2012a; Koc et al. 2013; Liebermann et al. 2014). Few studies have measured task performance before and after strabismus surgery. Lee et al. (2013) used a spatial localisation pointing task presented on a touch screen to measure pointing accuracy in patients pre- and post-XT surgery. Pointing accuracy was reduced one day postoperatively, but accuracy improved to preoperative levels at one month postoperatively (Lee et al. 2013).

#### Eye movements

Using a photoelectric eye tracker, Bucci et al. (2009) measured the accuracy and mean velocity of saccades, convergence and divergence, and combined saccades and vergence eye movements, pre- and postoperatively. Nine subjects (children and adults) with strabismus were included, six with no BSV pre- and postoperatively, although diplopia was not mentioned. Preoperatively, compared to normative data, accuracy was reduced for vergences and combined saccades and vergence; and mean velocity was reduced for saccades and convergence. Postoperatively, accuracy improved for saccades (at near), vergences and combined saccades and vergence; and mean velocity improved for convergence and combined saccades and divergence.

#### Limitations

Some studies included a heterogenous cohort and a wide range of patient ages. It is possible this may have introduced bias or variability in the interpretation of surgical outcome, particularly in studies where both children and adults were reported.

## Conclusion

Most of the evidence describing the outcomes of strabismus surgery in patients without visual symptoms reported improved postoperative ocular alignment and/or improved HRQoL. Yet, QoL and HRQoL measures were not used consistently, and different questionnaires were used. None of the questionnaires were exclusively for strabismus with psychosocial symptoms; however, the AS-20 was developed for adults with strabismus and was the most commonly used HRQoL questionnaire and PROM. There were variable reports of the outcomes of surgery, the time at which outcomes are measured and a lack of consensus on how success should be defined and measured after strabismus surgery. Strabismus surgery outcomes appeared to be measured satisfactorily at, or around, three months postoperatively. However, there is acknowledgement that the postoperative outcome at three months may differ from the longer-term outcome. Additional surgical outcomes, including an expanded field of vision, unexpected BSV, improved binocular summation, improved task performance and improved eye movements have been suggested, but have not been fully investigated. A core outcome set for strabismus has been suggested and there is potential to add to the available evidence by investigating which outcome measures are most relevant to those with strabismus and psychosocial symptoms. Criteria for ‘success, partial success, and failure’ have been used by several studies attempting to categorise and compare surgical outcomes. However, there is the potential to improve these categories, as patients categorised as a failure postoperatively can still report significant improvements postoperatively.

Overall, there was a lack of evidence specifically reporting the outcomes of strabismus surgery in adults with psychosocial symptoms. Large heterogeneous cohorts of strabismus patients were often reported, typically with a range of symptoms and differing surgical aims. There is a growing need for robust evidence in this specific subgroup of patients with strabismus and psychosocial symptoms.
